# A multicenter phase I/II study of enzalutamide in Japanese patients with castration-resistant prostate cancer

**DOI:** 10.1007/s10147-016-0952-6

**Published:** 2016-01-21

**Authors:** Hideyuki Akaza, Hirotsugu Uemura, Taiji Tsukamoto, Seiichiro Ozono, Osamu Ogawa, Hideki Sakai, Mototsugu Oya, Mikio Namiki, Satoshi Fukasawa, Akito Yamaguchi, Hiroji Uemura, Yasuo Ohashi, Hideki Maeda, Atsushi Saito, Kentaro Takeda, Seiji Naito

**Affiliations:** 1 Strategic Investigation On Comprehensive Cancer Network, The University of Tokyo, 4-6-1 Komaba, Meguro-ku, Tokyo, 153-8904 Japan; 2 Department of Urology, Kinki University Faculty of Medicine, 377-2 Ono-Higashi, Osakasayama, 589-8511 Japan; 3 Department of Urology, Sapporo Medical University School of Medicine, S1 W17 Chuo-ku, Sapporo, 060-8556 Japan; 4 Department of Urology, Hamamatsu University School of Medicine, 1-20-1 Handayama, Higashi-ku, Hamamatsu, 431-3192 Japan; 5 Department of Urology, Kyoto University Graduate School of Medicine, Yoshida-Konoe-cho, Sakyo-ku, Kyoto, 606-8501 Japan; 6 Department of Urology, Nagasaki University Graduate School of Biomedical Sciences, 1-7-1 Sakamoto, Nagasaki, 852-8501 Japan; 7 Department of Urology, Keio University School of Medicine, 35 Shinanomachi, Shinjuku-ku, Tokyo, 160-8582 Japan; 8 Department of Integrative Cancer Therapy and Urology, Kanazawa University Graduate School of Medical Sciences, 13-1 Takara-machi, Kanazawa, 920-8641 Japan; 9 Department of Urology, Chiba Cancer Center, 666-2 Nitona-Cho, Chuo-ku, Chiba, 260-8717 Japan; 10 Division of Urology, Harasanshin Hospital, 1-8 Taihakumachi, Hakata-ku, Fukuoka, 812-0033 Japan; 11 Department of Urology and Renal Transplantation, Yokohama City University Medical Center, 4-57 Urafune, Minami-ku, Yokohama, 232-0024 Japan; 12 Department of Integrated Science and Engineering for Sustainable Society, Chuo University, 1-13-27 Kasuga, Bunkyo-ku, Tokyo, 112-8551 Japan; 13 Astellas Pharma Inc, 2-5-1 Nihonbashi-Honcho, Chuo-Ku, Tokyo, 103-8411 Japan

**Keywords:** Androgen receptor inhibitor, Enzalutamide, Metastatic castration-resistant prostate cancer

## Abstract

**Background:**

The safety, tolerability, pharmacokinetics (PK) and anti-tumor activity of enzalutamide were investigated in patients with castration-resistant prostate cancer (CRPC) in Japan through a multicenter phase I/II study.

**Methods:**

In phase I, patients with progressive metastatic CRPC received single, then multiple, ascending doses of enzalutamide 80, 160 or 240 mg/day. After assessment of tolerability at multiple doses of 160 mg/day for 4 weeks, post-docetaxel patients with CRPC and measurable disease were enrolled into phase II; receiving long-term administration of enzalutamide 160 mg/day.

**Results:**

Nine and 38 patients were enrolled in phase I and II, respectively. During phase I, enzalutamide was well tolerated in each cohort; PK parameters were similar to those of non-Japanese populations in other studies. By week 12, overall response rate was 5.3 % and clinical benefit rate was 47.4 %. Prostate-specific antigen response rate (≥50 % reduction from baseline) was 28.9 %. Treatment-emergent adverse events reported in >20 % of patients in phase II were decreased weight, decreased appetite and constipation. No seizures were observed.

**Conclusion:**

Enzalutamide at 160 mg/day was well tolerated, with PK and safety profiles similar to the non-Japanese population. Anti-tumor activity was observed in post-docetaxel Japanese patients with metastatic CRPC. Apparent differences in anti-tumor activity compared with the AFFIRM study (a phase III trial in a diverse population of patients with CRPC post-docetaxel) may be attributed to differences in treatment history prior to starting enzalutamide. Particularly in Japan, the influence of sequence in hormone treatments, including combined androgen blockade therapy, should be considered.

Trial registration: ClinicalTrials.gov NCT01284920.

**Electronic supplementary material:**

The online version of this article (doi:10.1007/s10147-016-0952-6) contains supplementary material, which is available to authorized users.

## Introduction

As prostate cancer growth is dependent on androgens, androgen deprivation therapy (ADT), which includes surgical castration or medical therapy with gonadotropin-releasing hormone (GnRH) agonists or GnRH antagonists, is standard therapy for patients with metastatic prostate cancer recurrence after definitive therapy, or inoperable prostate cancer. In Japan, it is common practice in primary ADT to use androgen blockade combined with bicalutamide, a non-steroidal anti-androgen [1–4]. Progression of the disease despite castrate levels of testosterone under primary ADT is considered castration-resistant prostate cancer (CRPC) [5] and it generally represents a transition to the lethal state of the disease. CRPC is frequently treated with hormone therapy alternating with anti-androgens, low dose steroids or estrogenic compounds [6]. However, prolonged survival of patients with CRPC by these secondary hormonal treatments is not confirmed [7]. Until early 2014, docetaxel plus prednisone were the only approved drugs for patients with advanced CRPC in Japan [8, 9].

Recent treatment options that have demonstrated a survival improvement in patients with metastatic CRPC include cabazitaxel plus prednisone [10] and abiraterone plus prednisone [11, 12]. Enzalutamide [13, 14], sipuleucel-T [15] and radium Ra 223 dichloride [16] have also been approved for use in several countries.

Enzalutamide is a novel androgen receptor inhibitor that significantly prolongs survival of men with CRPC regardless of prior docetaxel therapy [13]. Enzalutamide inhibits multiple steps in the androgen receptor signaling pathway and is devoid of agonist activity in preclinical models [17]. Preclinical pharmacology studies have demonstrated that enzalutamide competitively inhibits androgen-induced receptor activation, nuclear translocation of activated androgen receptors, and the association of the activated androgen receptor with chromatin, even in the setting of androgen receptor over-expression and in prostate cancer cells resistant to anti-androgens [17].

The efficacy of enzalutamide was evaluated in two multinational phase III studies in men with metastatic CRPC; AFFIRM and PREVAIL. The AFFIRM trial showed overall survival (OS) benefit of enzalutamide in post-docetaxel patients with metastatic CRPC versus placebo. Median survival was 18.4 months with enzalutamide and 13.6 months with placebo [hazard ratio 0.63; 95 % confidence interval (CI) 0.53–0.75; *p* < 0.001] [14]. The PREVAIL trial confirmed clinical benefit of enzalutamide in chemotherapy-naïve patients with metastatic CRPC. The hazard ratio of OS and radiographic progression-free survival (rPFS) were 0.71 (95 % CI 0.60–0.84; *p* < 0.001) and 0.19 (95 % CI 0.15–0.23; *p* < 0.001), respectively [13]. Median OS was 32.4 months with enzalutamide and 30.2 months with placebo. Median rPFS was not reached with enzalutamide and was 3.9 months with placebo [13].

The present phase I/II clinical study evaluated the safety, tolerability and pharmacokinetics (PK) of enzalutamide in patients with CRPC and the anti-tumor activity and safety of enzalutamide in Japanese post-docetaxel patients with CRPC to provide supporting data for the regulatory approval of enzalutamide in Japan.

## Patients and methods

### Study design

This was a multicenter, open-label, uncontrolled study of orally administered enzalutamide, involving two phases (http://ClinicalTrials.gov NCT01284920). Phase I involved dose escalation in patients with CRPC. Phase II involved dose expansion in post-docetaxel patients with CRPC.

All participating sites obtained approval for conducting the study by their institutional review boards. The study was conducted in accordance with the Declaration of Helsinki, Good Clinical Practice Guidelines and the Pharmaceutical Affairs Law in Japan. All patients provided written informed consent to participate in the study.

In phase I, patients received a single dose of enzalutamide (80, 160 or 240 mg/day) and blood samples for PK analysis were collected over 7 days. Subsequently, patients received multiple doses of enzalutamide at the same dosage levels as in the single-dose period. Tolerability was evaluated 29 days after initiation of repeat dosing by an independent data monitoring committee. Patients who received 240 mg in the single-dose period subsequently received multiple doses of 160 mg/day (the recommended dose in the AFFIRM study [12]). Following the evaluation of enzalutamide tolerability and PK parameters after single and multiple doses at 160 mg in phase I, an open-label, uncontrolled phase II study was initiated to evaluate the efficacy, safety and PK in patients receiving enzalutamide 160 mg/day. The study design was discussed with the Pharmaceuticals and Medicine Devices Agency from a perspective of regulatory approval of enzalutamide in Japan. Consequently, overall response rate was selected to be the primary outcome variable in this study, thereby requiring enrolment of patients with measureable disease into the study.

### Patients

Patients with metastatic CRPC who had disease progression while on castration therapy were eligible for participation. Patients had to have received ADT with a GnRH analogue or a bilateral orchiectomy with serum testosterone level maintained within castration level (≤50 ng/dL).

The criteria used to define disease progression for trial entry are available in the Online Resource. Eligible patients had Eastern Cooperative Oncology Group performance status of 0 or 1 (or 2 if only due to metastatic bone pain at the screening). Post-chemotherapy patients had to have received prior chemotherapy with docetaxel and no more than two prior chemotherapy regimens. In particular for phase II, patients had to have measurable lesions as determined by response evaluation criteria for solid tumors (RECIST) guidelines.

Exclusion criteria were history of seizure (including any febrile seizure, loss of consciousness or transient ischemic attack within 12 months prior to initiation of study drug) or any condition that may predispose to seizure. The complete list of exclusion criteria is available in the Online Resource.

### Assessments

The primary outcome in anti-tumor activity in phase II was best overall response by 12 weeks; defined by RECIST guidelines as complete response (CR) or partial response (PR) and assessed by an investigator. Confirmation of CR or PR was required by a subsequent scan at least 4 weeks later. When the investigator confirmed CR or PR, the assessment was finally evaluated by an independent RECIST assessment committee. Measurements had to meet the stable disease criteria by day 85 for determination of stable disease. Radiographic imaging for the target region was conducted at the screening visit, on day 29, day 57 and day 85, and at each subsequent visit every 84 days. Bone scans were examined at the screening visit and at each 84-day visit.

The secondary endpoint was prostate-specific antigen (PSA) response rate (proportion of subjects with ≥50 % decline in serum PSA from baseline). Serum PSA measurements were conducted at the screening visit and at each subsequent visit every 28 days.

Safety was evaluated from the start of study treatment to 30 days after completion of the study treatment. All adverse events (AEs) were recorded using the National Cancer Institute Common Terminology Criteria for Adverse Events (NCI CTCAE), version 4.0, and Medical Dictionary for Regulatory Activities (MedDRA), version 14.1. Laboratory values, vital signs, body weight and 12-lead echocardiograms were assessed at predefined time points.

Blood samples were collected at predefined time points in phase I and phase II. Plasma concentrations of enzalutamide and its active metabolite, *N*-desmethyl enzalutamide, were determined by a validated bioanalytical method based on liquid chromatography combined with mass spectrometry [18]. PK parameters were estimated by non-compartmental methods in WinNonlin^®^ (Pharsight Corp., Palo Alto, CA, USA) and included maximum plasma concentration (C_max_) and area under the plasma concentration–time curve from time 0 to infinity (AUC_∞_). To investigate potential PK differences between Japanese and non-Japanese patients, the PK data were compared with PK data from AFFIRM.

### Statistical analysis

The number and percentage of patients with a best overall response by day 85 and two-sided 90 and 95 % Clopper–Pearson CIs were used in the primary analysis. A waterfall plot of maximum percent change from baseline of serum PSA was created. The number and percentage of patients with best PSA response at time of nadir were summarized. All data processing, summarization and analyses were performed using SAS Drug Development, version 3.4, and PC-SAS, version 9.1.3. All analyses were performed by the sponsor using data obtained by the cut-off date of 12 July 2012.

### Post hoc analysis

An additional post hoc exploratory analysis was conducted to further compare enzalutamide anti-tumor activity in Japanese patients with non-Japanese patients with measurable disease from the AFFIRM study. The best overall response by number of prior hormonal therapy lines, and defined by RECIST and PSA response rate, was calculated.

## Results

### Patients

In phase I, three patients were assigned to each of the 80, 160 and 240 mg groups. The median duration of exposure in each group was 584.0, 171.0 and 252.0 days, respectively. Thirty-eight post-docetaxel patients with CRPC and measurable disease as defined by RECIST were enrolled into phase II at a dose of 160 mg/day. Median duration of exposure was 121 days. No remarkable differences were observed in the demographic and clinical baseline characteristics between the phase I and phase II study populations (Table 1). Patients in phase II were heavily pretreated, with >90 % having had ≥4 prior hormonal treatments (Table 2). Eight of 38 patients (21.1 %) in phase II discontinued due to AEs. Of these, five patients withdrew due to disease progression (Table 3). All patients had received complete androgen blockade (CAB) therapy with bicalutamide soon after their initial diagnosis of prostate cancer. Overall, 42.1 % had >10 bone metastases and all patients had measurable disease by RECIST (Table 1).

**Table 1:** Summary of demographics and other baseline characteristics

Demographic/characteristic	Phase I (*N* = 9)	Phase II (*N* = 38)
Age (years)		
Median	73.0	71.5
Min–max	62–86	50–85
Height (cm)		
Median	166.0	165.7
Min–max	156.2–174.4	153.4–181.0
Weight (kg)		
Median	71.2	65.7
Min–max	49.2–88.9	49.2–93.0
ECOG PS^a^		
Grade 0	8 (88.9)	25 (65.8)
Grade 1	1 (11.1)	13 (34.2)
Total Gleason score^b^ at initial diagnosis^a^		
Low, 2–4	0	0
Medium, 5–7	0	8 (21.1)
High, 8–10	9 (100.0)	29 (76.3)
Unknown	0	1 (2.6)
Clinical tumor stage (T)^c^ at initial diagnosis^a^		
TX	1 (11.1)	1 (2.6)
T0	0	0
T1	0	0
T2	1 (11.1)	10 (26.3)
T3	6 (66.7)	16 (42.1)
T4	1 (11.1)	10 (26.3)
Unknown	0	1 (2.6)
Clinical lymph node stage at initial diagnosis^a^		
NX	2 (22.2)	1 (2.6)
N0	3 (33.3)	14 (36.8)
N1	4 (44.4)	22 (57.9)
Unknown	0	1 (2.6)
Distant metastasis (M)^c^ at initial diagnosis^b^		
MX	0	1 (2.6)
M0	3 (33.3)	17 (44.7)
M1	6 (66.7)	19 (50.0)
Unknown	0	1 (2.6)
Number of bone metastases		
0	3 (33.3)	8 (21.1)
1	1 (11.1)	1 (2.6)
2–4	1 (11.1)	6 (15.8)
5–9	0	7 (18.4)
≥10	4 (44.4)	16 (42.1)
Anti-androgen withdrawal syndrome^a^		
Yes	0	4 (10.5)
Stage of prostate cancer^a,b^		
Localized	1 (11.1)	6 (15.8)
Locally advanced	2 (22.2)	11 (28.9)
Metastatic	6 (66.7)	19 (50.0)
Not classifiable	0	2 (5.3)
PSA at baseline (ng/mL)		
Mean (SD)	634.82 (1403.52)	174.94 (307.97)
Median	21.60	65.80
Duration of disease at screening (months)		
Mean (SD)	47.36 (22.23)	63.11 (38.15)
Median	39.93	52.83

*ECOG PS* Eastern Cooperative Oncology Group performance status, *PSA* prostate-specific antigen, *SD* standard deviation

^a^Number (%) of patients

^b^Gleason [27]

^c^Classified using the TNM classification [28] as follows: localized, T1/2 and (NX or N0) and M0; locally advanced, T3/4 and (NX or N0) and M0 or N1 and M0; metastatic, M1; Not classifiable, others

**Table 2:** Prior treatments for prostate cancer in phase II

Parameter	Category/statistic	Phase II (*N* = 38)
Cancer treatment history, radiation	Yes	19 (50.0 %)
Cancer treatment history, procedure	Yes	6 (15.8 %)
Quantity of prior hormone therapy lines^a^	3	3 (7.9 %)
	4	5 (13.2 %)
	5	13 (34.2 %)
	6	11 (28.9 %)
	≥7	6 (15.8 %)
Typical prior hormone therapy, other than GnRH analogue	Bicalutamide	38 (100.0 %)
	Flutamide	29 (76.3 %)
	Estramustine	30 (78.9 %)
	Docetaxel	38 (100 %)
Number of prior chemotherapy regimens	1	8 (21.1 %)
	2	30 (78.9 %)
Duration of prior docetaxel (days)	Median	198
	Min–max	1–1012

*GnRH* gonadotropin-releasing hormone

^a^Sum of prior hormonal treatment agents including castration therapy

**Table 3:** Primary reasons for discontinuation

Category and reason	Phase I (*N* = 9)	Phase II (*N* = 38)
Discontinuation in multiple-dose period (early termination), *n* (%)		
Adverse event	0	5 (13.2)
Worsening of disease	1 (11.1)	5 (13.2)
Withdrawal by subject	0	2 (5.3)
Discontinuation in overall study, *n* (%)		
Adverse event	0	8 (21.1)
Worsening of disease	5 (55.6)	18 (47.4)
Withdrawal by subject	0	2 (5.3)

### Anti-tumor activity

#### Response

The best overall response rate (CR and PR) by day 85, as evaluated by the RECIST assessment committee and investigators, was seen in 5.3 % of patients (two out of 38; 95 % CI 0.6–17.7 %; 90 % CI 0.9–15.7 %). The best overall disease control rate (CR plus PR plus stable disease) by day 85 was 47.4 % of patients (18 out of 38; 95 % CI 31.0–64.2 %; 90 % CI 33.3–61.8 %) (Table 4). The rate with which the sum of diameters of target lesions was reduced by ≥30 % was 18.4 % (7 out of 38 patients).

**Table 4:** Best overall responses by day 85

Best overall response	Evaluation by RECIST assessment committee and investigator^a^ (*N* = 38)
CR, *n*	0
PR, *n* (%)	2 (5.3)
Stable disease, *n* (%)	16 (42.1)
PD, *n* (%)	16 (42.1)
Not evaluated	4 (10.5)
CR or PR, *n* (%) (response rate)	2 (5.3)
95 % CI^b^	0.6–17.7 %
90 % CI^b^	0.9–15.7 %
CR or PR or stable disease, *n* (%) (disease control rate)	18 (47.4)
95 % CI^b^	31.0–64.2 %
90 % CI^b^	33.3–61.8 %

Tumor response (overall response) for each patient was assessed by the investigator and subsequently evaluated by an independent RECIST assessment committee (when the investigator assessed that a patient had been accomplished CR or PR)

*CR* complete response, *PD* progressive disease, *PR* partial response

^a^When there were evaluation data from both the RECIST committee and investigator, RECIST assessment committee data were adopted

^b^Based on exact binomial confidence interval (Clopper–Pearson)

#### PSA

**Fig. 1:**
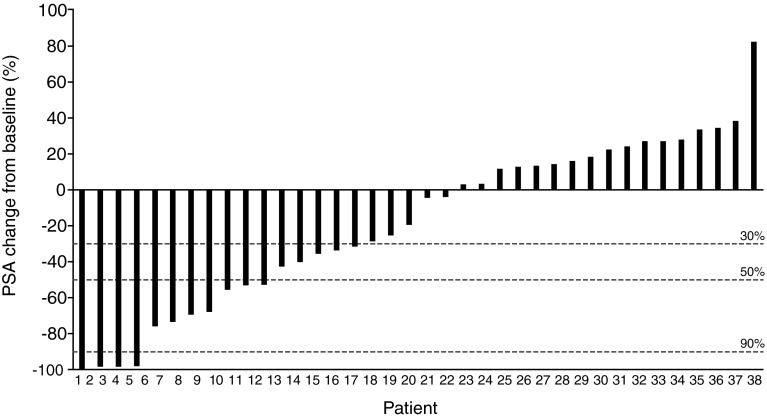
Waterfall plot of maximum percent change from baseline of serum PSA in phase II. *PSA*, prostate-specific antigen

Eleven out of 38 patients in phase II (28.9 %; 95 % CI 15.4–45.9 %) had a ≥50 % decrease in PSA levels at the time of nadir, as compared with baseline (Fig. 1; Table 5).

**Table 5:** Best PSA response at time of nadir

Response	160 mg/day (*N* = 38)
Decline from baseline, patients, *n* (%)	
≥ 30 %	15 (39.5)
95 % CI	24.0–56.6 %
≥50 %	11 (28.9)
95 % CI	15.4–45.9 %
≥90 %	4 (10.5)
95 % CI	2.9–24.8 %

*CI* confidence interval, *PSA* prostate-specific antigen

### Safety

The most frequent treatment-emergent AEs (TEAEs) with an incidence of ≥20 % across both phases were weight decrease (36.2 %), decreased appetite (27.7 %) and constipation (25.5 %) (Table 6). Of the adverse drug reactions reported in ≥10 % of patients, those considered to be related to the study drug were hypertension (14.9 %), constipation (14.9 %), fatigue (12.8 %), decreased appetite (12.8 %), weight decrease (10.6 %) and electrocardiogram QT prolonged (10.6 %). None of the TEAEs resulted in death and no seizures were reported. The most common serious TEAE was cancer pain (*N* = 3) (Table 7).

**Table 6:** Common adverse events (reported in at least 10 % of patients in total)

MedDRA, version 14.1, preferred term	All adverse events			Adverse events considered to be related to study drug		
	Phase I (*N* = 9)	Phase II (*N* = 38)	Total (*N* = 47)	Phase I (*N* = 9)	Phase II (*N* = 38)	Total (*N* = 47)
Overall	9 (100.0)	36 (94.7)	45 (95.7)	7 (77.8)	24 (63.2)	31 (66.0)
Weight decreased	1 (11.1)	16 (42.1)	17 (36.2)	0	5 (13.2)	5 (10.6)
Decreased appetite	3 (33.3)	10 (26.3)	13 (27.7)	2 (22.2)	4 (10.5)	6 (12.8)
Constipation	2 (22.2)	10 (26.3)	12 (25.5)	1 (11.1)	6 (15.8)	7 (14.9)
Hypertension	3 (33.3)	6 (15.8)	9 (19.1)	3 (33.3)	4 (10.5)	7 (14.9)
Cancer pain	1 (11.1)	8 (21.1)	9 (19.1)	0	1 (2.6)	1 (2.1)
Nausea	4 (44.4)	5 (13.2)	9 (19.1)	1 (11.1)	2 (5.3)	3 (6.4)
Electrocardiogram QT prolonged	0	6 (15.8)	6 (12.8)	0	5 (13.2)	5 (10.6)
Fatigue	2 (22.2)	4 (10.5)	6 (12.8)	2 (22.2)	4 (10.5)	6 (12.8)
Nasopharyngitis	1 (11.1)	5 (13.2)	6 (12.8)	0	0	0
Pyrexia	1 (11.1)	4 (10.5)	5 (10.6)	1 (11.1)	0	1 (2.1)
Somnolence	0	5 (13.2)	5 (10.6)	0	1 (2.6)	1 (2.1)
Rash	0	5 (13.2)	5 (10.6)	0	1 (2.6)	1 (2.1)

Number of patients (%)

*MedDRA* Medical Dictionary for Regulatory Activities

**Table 7:** Serious treatment-emergent adverse events (with an incidence of ≥2 events in the study)

MedDRA, version 14.1, preferred term	Phase I total^a^ (*N* = 9)	Phase II 160 mg (*N* = 38)
Overall	2 (22.2)	13 (34.2)
Cancer pain	1 (11.1)	2 (5.3)
Anemia	0	2 (5.3)
Disseminated intravascular coagulation	0	2 (5.3)
General physical health deterioration	0	2 (5.3)
Cellulitis	0	2 (5.3)
Tumor pain	0	2 (5.3)
Bladder tamponade	0	2 (5.3)

Number of patients (%)

*MedDRA* Medical Dictionary for Regulatory Activities

^a^In phase I, safety data from single doses (80, 160 and 240 mg) and multiple doses (80 and 160 mg) are included. All patients in the 240 mg group received enzalutamide at a dose of 160 mg after single dosing

### Pharmacokinetics

**Fig. 2:**
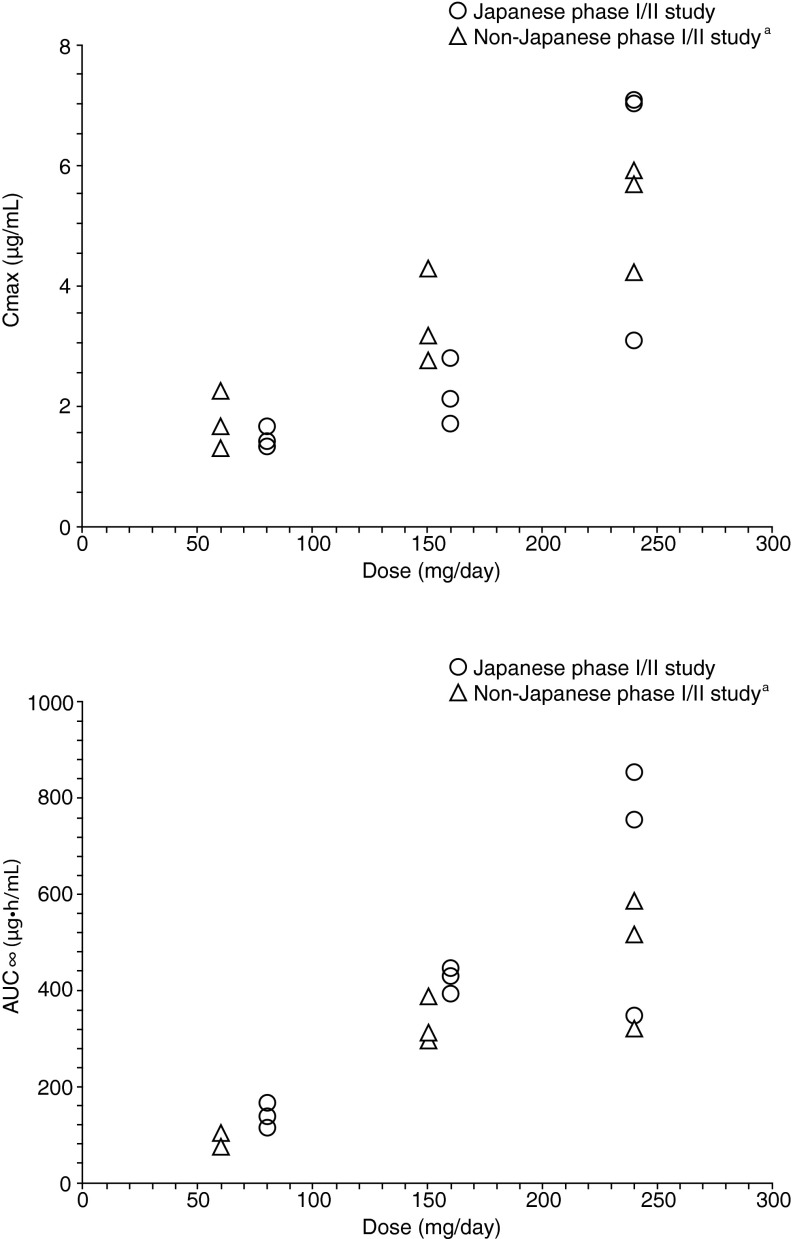
Comparison of individual enzalutamide C_max_ and AUC_∞_ during single-dosing. ^a^Phase I, open-label, dose-escalation safety and pharmacokinetic study of enzalutamide in patients with CRPC conducted overseas [19]

**Fig. 3:**
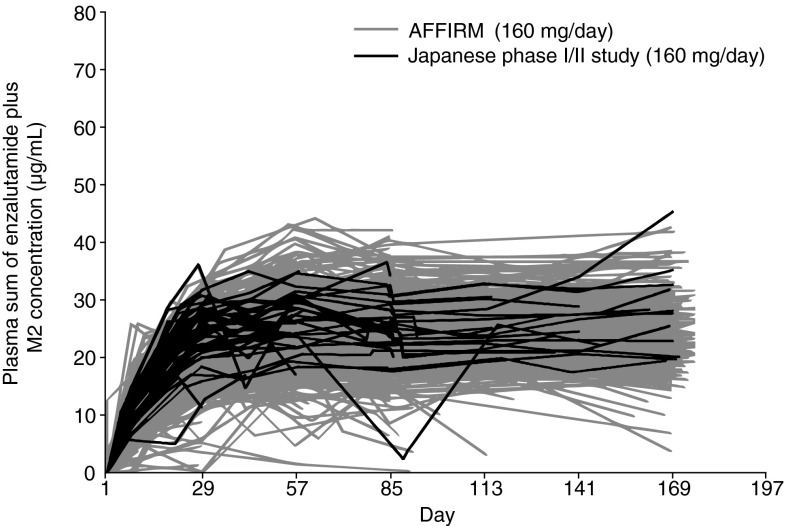
Individual trough plasma sum of enzalutamide and active metabolite concentration versus time plot in the Japanese phase I/II and AFFIRM (International, phase III, randomized, double-blind, placebo-controlled study of enzalutamide in patients with prostate cancer who had previously been treated with one or two chemotherapy regimens, at least one of which contained docetaxel [14]) studies, up to day 169

Enzalutamide was absorbed rapidly after oral administration in Japanese patients and the PK was dose-proportional after a single dose ranging from 80 to 240 mg (Fig. 2). The PK profile of a single dose of enzalutamide in Japanese patients was similar to that of non-Japanese patient data from the first enzalutamide study in humans (phase I/II study; http://ClinicalTrials.gov NCT00510718) (Fig. 2) [19]. PK profiles of the sum of enzalutamide plus its active metabolite in plasma were similar between Japanese patients from the current study and non-Japanese populations from AFFIRM (Fig. 3).

### Post hoc analysis

An exploratory post hoc analysis compared the subgroup of non-Japanese patients with measurable disease from AFFIRM [*N* = 446 out of 800 enzalutamide-treated patients (cut-off date: 25 September 2011)] and Japanese patients from the current study (*N* = 38). The quantity of prior hormonal therapy lines used as prostate cancer treatment in the two studies, which excluded medical or surgical castration therapy, is available in the Online Resource in Table S1. While approximately 90 % of patients in the AFFIRM study had received ≤2 hormonal therapy lines, approximately 90 % of patients in the current study had received ≥3 hormonal therapy lines. Best overall response rate, by RECIST, and PSA response rate for each amount of prior hormonal therapy lines are available in the Online Resource in Table S2 and Table S3, respectively.

## Discussion

In patients who receive primary androgen deprivation therapy, the proportion of patients with high risk and/or advanced prostate cancer is higher in Japan than in the United States [2]. A randomized, controlled study of primary ADT by CAB with chemical castration by GnRH agonist and bicalutamide 80 mg in Japanese patients showed significant prolongation of OS compared with chemical castration alone [20]. Based on this result, CAB is used in Japan as a standard initial therapy for high-risk or progressive prostate cancer [2, 4].

Results from this first clinical study of enzalutamide in the Japanese post-docetaxel CRPC patient population showed that enzalutamide was well tolerated when orally administered at a dose of 160 mg once daily. PK of enzalutamide was dose-proportional in the doses ranging from 80 to 240 mg and similar to PK data from non-Japanese patients. Furthermore, enzalutamide administered orally at 160 mg once daily had anti-tumor activity in Japanese post-chemotherapy patients with CRPC, in terms of best overall response or tumor-shrinking tendency and PSA response.

However, this study did not achieve radiographic and PSA response rates as high as those in AFFIRM. The radiographic response rate by day 85 was 5.3 % in the current study versus 28.9 % in AFFIRM. The PSA response rate (≥50 % reduction from baseline) was 54.0 % in AFFIRM, compared with 28.9 % in this study. Differences between the two studies (i.e., patient populations enrolled, study setting and design, patient samples in each trial) may account for the lower radiographic and PSA response rates. As prior docetaxel exposure received by patients in this study (median, 198 days; approximately 9–10 cycles) was similar to that reported by patients in the AFFIRM trial (median, 8.5 cycles) [14], the most important difference could be that patients in the current study had received more hormonal therapy lines prior to enzalutamide compared with those in AFFIRM. With the exclusion of castration therapies, approximately 90 % of patients in this study had received ≥3 prior hormonal therapy lines (i.e., CAB, anti-androgen alternative therapy, steroids, and estrogens), whereas patients typically received ≤2 lines in AFFIRM. This difference may be related to the recommended treatment strategy in Japan, which includes extensive exposure to CAB, with bicalutamide 80 mg as the primary ADT and further treatment with alternating hormone therapy. This observation is also supported by the results of a previous surveillance study by the Japan Study Group of Prostate Cancer (J-CaP) that considered the current status of endocrine therapy for prostate cancer [4]. Of the 3337 patients who initially received primary ADT, 2477 patients (74.2 %) were given CAB in the J-Cap surveillance [4]. The pattern of primary ADT usage was more common in Japan than in the United States and primary ADT by CAB was associated with better survival than other forms of primary ADT in Japanese high-risk patients [2]. Although extensive direct comparisons between Japan and the United States are not possible, there are some differences between the two countries in the initial prostate cancer treatment selection and outcome [2, 4].

Furthermore, although hormonal treatments have been the mainstay of treatment in advanced prostate cancer, recent data suggest potential development of cross-resistance after multiple lines of hormonal therapy [21, 22]. In the first-in-man enzalutamide phase I/II study in patients in the United States, the rate of PSA decline of ≥50 % was significantly lower in patients with previous ketoconazole treatment versus those without [37 % (95 % CI 25–50 %) versus 71 % (95 % CI 60–81 %; *p* = 0.0007)] [19]. Moreover, a study of abiraterone acetate plus prednisone showed that patients with prior exposure to ketoconazole had a lower percentage of PSA decline of ≥50 % compared with ketoconazole-naïve patients [23]. In the same study, time to PSA progression was shorter in patients with prior ketoconazole exposure compared with ketoconazole-naïve patients [23]. In addition, there have been several recent reports on cross-tolerance between abiraterone plus prednisone and enzalutamide. Reports from compassionate use programs involving heavily pretreated patients with metastatic CRPC suggest reduced efficacy for both enzalutamide and abiraterone in comparison to the efficacy reported in clinical trials [24, 25]. Furthermore, results of a recent study in 32 patients suggested a potential effect of androgen receptor splice variant-7 on primary treatment resistance, observed with abiraterone plus prednisone or enzalutamide [26].

Enzalutamide showed good tolerability in Japanese patients, with PK and safety profiles similar to those in non-Japanese populations included in other enzalutamide studies. The differences in anti-tumor activity observed in this study versus the AFFIRM trial may be attributed to differences in the study design and patients’ backgrounds in each trial. In particular, they may be attributed to differences in treatment history prior to starting enzalutamide. This may require further investigation to define the optimal timing and treatment strategy of enzalutamide for patients with CRPC. Particularly in Japan, the influence of sequence for hormone treatments, including CAB therapy, should be considered.

## Electronic supplementary material

Below is the link to the electronic supplementary material.

Supplementary material 1 (DOCX 22 kb)
