# Supplementary material for: A multicenter phase I/II study of enzalutamide in Japanese patients with castration-resistant prostate cancer
Source: Int J Clin Oncol. 2016 Jan 21;21:773–82. doi: 10.1007/s10147-016-0952-6 (PMC4967591; doi:10.1007/s10147-016-0952-6)
Supplement: Supplementary file 1 — Supplementary material 1 (DOCX 22 kb) [file 10147_2016_952_MOESM1_ESM.docx]

**A multicenter phase I/II study of enzalutamide in Japanese patients with castration-resistant prostate cancer who had received combined androgen blockade**

Hideyuki Akaza et al

**Supplementary material**

**Disease progression criteria**

**Additional exclusion criteria**

**Table S1**

**Table S2**

**Table S3**

**Disease progression criteria**

Progression of prostate cancer disease at trial entry was defined by one or more of the following criteria: soft tissue progression according to Response Evaluation Criteria for Solid Tumors, version 1.1; prostate-specific antigen (PSA) progression as defined by three consecutive rising PSA levels with an interval of ≥1 week between each determination with absolute PSA level ≥2 ng/mL; or bone progression with at least two or more new lesions on bone scan during the pretreatment of the study, according to Prostate Cancer Clinical Trials Working Group recommendations [1].

**Additional exclusion criteria**

Patients with metastases in the brain, history of a malignancy other than prostate within 5 years, use of bicalutamide within 6 weeks prior to study initiation, use of hormonal treatment other than bicalutamide within 4 weeks prior to study initiation, use of steroids at doses greater than the equivalent of 10 mg/day of prednisolone within 4 weeks prior to study initiation and radiation therapy within 12 weeks prior to study initiation were excluded. Patients were also excluded by presence of high-grade abnormalities in laboratory findings.

Table S1 Number of prior hormonal therapy lines as treatment for prostate cancer

| Parameter | Category | Phase I/II Japanese patients  (*N* = 38) | Phase III AFFIRM non-Japanese patients  (*N* = 446) |
| --- | --- | --- | --- |
| Number of hormonal therapy lines^a^ | 0 | 0 | 29 (6.5 %) |
|  | 1 | 0 | 218 (48.9 %) |
|  | 2 | 4 (10.5 %) | 141 (31.6 %) |
|  | 3 | 5 (13.2 %) | 44 (9.9 %) |
|  | ≥4 | 29 (76.3 %) | 14 (3.1 %) |

^a^Hormonal therapies include anti-androgen agents, estramustine phosphate sodium hydrate, corticosteroids and estrogen, but not gonadotropin-releasing hormone analogues

Table S2 Response rate of best overall response, by RECIST, by number of prior hormonal therapy lines

| Outcome | Number of hormonal therapy lines (except GnRH analogues) | Phase I/II  Japanese patients  (*N* = 38)^a^ | Phase III AFFIRM non-Japanese patients  (*N* = 446) |
| --- | --- | --- | --- |
| Response rate**^b^** | 0 | 0 | 11/29 (37.9 %) |
|  | 1 | 0 | 61/218 (28.0 %) |
|  | 2 | 0/4 (0.0 %) | 45/141 (31.9 %) |
|  | 3 | 1/5 (20.0 %) | 10/44 (22.7 %) |
|  | ≥4 | 1/29 (3.4 %) | 2/14 (14.3 %) |

**^a^**Best overall response by day 85. If there were two different assessments by the investigator and by the assessment committee members, the assessment by the assessment committee members was prioritized; **^b^**Response rate: proportion of patients achieving complete or partial response

*GnRH* gonadotropin-releasing hormone, *RECIST* Response Evaluation Criteria for Solid Tumors

Table S3 PSA response rate by number of prior hormonal therapy lines

| Outcome | Number of hormonal therapy lines (except GnRH analogues) | Phase I/II  Japanese patients  (*N* = 38) | Phase III AFFIRM non-Japanese patients (*N* = 392) |
| --- | --- | --- | --- |
| PSA response rate^a^ | 0 | 0 | 15/23 (65.2 %) |
|  | 1 | 0 | 107/195 (54.9 %) |
|  | 2 | 0/4 (0.0 %) | 70/120 (58.3 %) |
|  | 3 | 1/5 (20.0 %) | 18/40 (45.0 %) |
|  | ≥4 | 10/29 (34.5 %) | 5/14 (35.7 %) |

^a^Proportion of patients who had a ≥50 % reduction from baseline in prostate-specific antigen during the evaluation period

*GnRH* gonadotropin-releasing hormone, *PSA* prostate-specific antigen

**References**

1. Ryan CJ, Smith MR, de Bono JS, et al (2013) Abiraterone in metastatic prostate cancer without previous chemotherapy. N Engl J Med 368:138–148
